# A Comparative American Board of Surgery In-Training Examination (ABSITE) Performance Analysis Between International vs. Domestic Graduates and Doctor of Medicine (MD) vs. Doctor of Osteopathic Medicine (DO) Medical Degrees

**DOI:** 10.7759/cureus.62358

**Published:** 2024-06-14

**Authors:** Armein Rahimpour, Missy Morrison, David A Denning, Paul Bown, Peter Ray, Rahman Barry

**Affiliations:** 1 General Surgery, Marshall University Joan C. Edwards School of Medicine, Huntington, USA; 2 Plastic Surgery, UK King's Daughters Medical Center, Ashland, USA; 3 Plastic and Reconstructive Surgery, Marshall University Joan C. Edwards School of Medicine, Huntington, USA

**Keywords:** truelearn, american board of surgery, international medical school graduate, general surgery residency training, american board of surgery in-training examination

## Abstract

Introduction

The American Board of Surgery (ABS) plays a pivotal role in certifying surgeons in the United States, with the American Board of Surgery In-Training Examination (ABSITE) serving as a critical assessment tool for general surgery residents aspiring for certification. The aim of this study is to compare the performance of international medical graduates (IMGs) to their domestic counterparts and assess the impact of different medical degrees on ABSITE scores. Notably, ABSITE scores often dictate the trajectory of a surgical career, including opportunities for fellowship placements in specialized fields such as plastic surgery.

Methods

This study focused on general surgery residents enrolled at Marshall University from 2014 to 2022. Data encompassing ABSITE scores, TrueLearn quiz percentages, and TrueLearn mock exam results were collected for analysis. Descriptive statistics summarized sample characteristics, and linear mixed models were employed to address correlations. Statistical analyses were conducted using the Statistical Analysis System (SAS) (version 9.4; SAS Institute Inc., Cary, NC, USA), with significance defined by a two-sided test with p < 0.05.

Results

Among the 48 participants, comprising 24 non-international medical graduates (nIMGs) and 24 IMGs, IMGs demonstrated superior performance across various metrics. They exhibited higher quiz percentages (67% vs. 61%; p = 0.0029), mock Exam 1 scores (64% vs. 58%; p = 0.0021), mock Exam 2 scores (66% vs. 58%; p = 0.0015), ABSITE scores (560 vs. 505; p = 0.010), and ABSITE percentages (74% vs. 68%; p = 0.0077) compared to nIMGs. Analysis between Doctor of Osteopathic Medicine (DO) and Doctor of Medicine (MD) participants revealed no statistically significant differences in performance metrics, highlighting the comparability of these medical degrees in the context of ABSITE scores and related assessments.

Discussion/conclusion

This study underscores the superior performance of IMGs over nIMGs in ABSITE examinations, shedding light on the critical role of ABSITE scores in shaping surgical careers. Higher scores correlate with enhanced opportunities for coveted fellowship placements, particularly in specialized fields like plastic surgery. Understanding these dynamics is crucial for resident training and navigating the competitive landscape of surgical sub-specialization. Future research endeavors can delve deeper into the factors influencing ABSITE performance, thereby facilitating the development of targeted interventions to support residents in achieving their career aspirations.

## Introduction

The American Board of Surgery (ABS) stands as a cornerstone institution in the certification and credentialing of surgeons across the United States, wielding profound influence over the trajectories of surgical careers through its rigorous assessment protocols, notably including the American Board of Surgery In-Training Examination (ABSITE). This pivotal examination serves as a litmus test for general surgery residents, offering a comprehensive evaluation of their knowledge and readiness for the challenges of surgical practice, while simultaneously shaping their future opportunities for specialization, research, and professional advancement [1].

Against this backdrop of stringent evaluation and career-defining milestones, the performance differentials among diverse cohorts of surgical trainees have emerged as a subject of considerable interest and scrutiny within the medical community. Particularly, the comparative analysis of international medical graduates (IMGs) and their domestic counterparts has garnered attention due to its implications for workforce diversity, training efficacy, and healthcare equity [2,3]. As such, investigating the ABSITE performance of IMGs vis-à-vis domestic graduates constitutes a salient research endeavor, offering insights into the efficacy of training programs, the impact of cultural and educational backgrounds on medical competencies, and the broader landscape of surgical education and practice in an increasingly globalized world [4].

Furthermore, the ABSITE, beyond its immediate function as an evaluative tool, holds significant sway over the career trajectories of surgical residents, exerting influence over fellowship placements, career opportunities, and professional standing within the surgical community [5]. Given the highly competitive nature of surgical subspecialties and the stringent selection criteria imposed by fellowship programs, ABSITE scores assume heightened importance as a determinant of future career pathways, particularly in coveted fields such as plastic surgery, where specialization demands exceptional proficiency and expertise [5].

Amidst these considerations, this study endeavors to elucidate the performance differentials between IMGs and domestic graduates in ABSITE examinations, with a view towards delineating the factors that underpin such disparities and assessing their implications for surgical training, workforce diversity, and career progression within the realm of general surgery. By contextualizing ABSITE performance within the broader landscape of surgical education and professional development, this research seeks to inform evidence-based interventions, policy reforms, and educational initiatives aimed at fostering inclusivity, equity, and excellence within the surgical workforce. Through rigorous analysis and empirical inquiry, this study endeavors to contribute to the ongoing discourse surrounding surgical education, training methodologies, and the cultivation of a diverse, competent, and resilient cadre of surgical practitioners equipped to meet the multifaceted challenges of contemporary healthcare delivery.

## Materials and methods

The research protocol for this study, ethically approved by the Marshall University Institutional Review Board (IRB No. 2097669-1), granted authorization for data collection and analysis, focusing on assessing the performance of general surgery residents enrolled at Marshall University from 2014 to 2022. The inclusion criteria encompassed all residents working at the hospital, including both categorical and preliminary residents. No residents were excluded, as no exclusion criteria were applied. The study utilized data from TrueLearn, a comprehensive educational platform extensively used in medical training and preparation for standardized examinations such as the ABSITE. TrueLearn offers a suite of practice resources, including quizzes and mock exams, designed to replicate real assessment conditions, providing learners with valuable opportunities to reinforce their knowledge, identify areas for improvement, and gauge their readiness for high-stakes examinations.

The data collection process involved centralizing information using Microsoft Excel software (Microsoft® Corp., Redmond, WA, USA). Over a span of nine years, data for each year were organized into separate Excel sheets. To maintain confidentiality, resident names were anonymized and replaced with numerical identifiers. ABSITE scores were obtained directly from the ABS. Additionally, TrueLearn was contacted to gather supplementary data, including quiz percentages, as well as results from mock exams 1 and 2, for each resident.

Comprehensive datasets were compiled, encompassing ABSITE scores, Quiz %, Exam 1, and Exam 2. The "Quiz %" reflects the overall percentage of correct responses obtained in quizzes administered via the TrueLearn platform, while "Exam 1" and "Exam 2" denote the initial and subsequent mock examinations undertaken within the TrueLearn system, respectively. Quiz percentages and exams were chosen as the key variables for this study due to their availability as ABSITE preparation resources provided by TrueLearn to residents. Additionally, participation in mock exams and quizzes is mandatory for residents at Marshall University. Additionally, pertinent details regarding residents' graduation status were meticulously documented, distinguishing between IMGs and non-international medical graduates (nIMGs), along with their degree type, differentiating between Doctor of Medicine (MD) and Doctor of Osteopathic Medicine (DO).

Descriptive statistics were used to summarize the sample characteristics. Continuous variables were expressed as means ± standard deviations (SD). The data were collected repeatedly over multiple years for the sample. All statistical analyses were conducted using the Statistical Analysis System (SAS) (version 9.4; SAS Institute Inc., Cary, NC, USA). Statistical significance was defined using a two-sided test with a p-value < 0.05.

## Results

The study included a total of 48 general surgery residents (Table 1). Among the participants, 24 were nIMGs, and the remaining 24 were IMGs. IMGs demonstrated higher quiz percentages compared to nIMGs (67% vs. 61%; β = 7.29, 95% CI [2.58, 12], p = 0.0029). Similarly, they outperformed nIMGs in mock Exam 1 (64% vs. 58%; β = 7.43, 95% CI [2.79, 12.06], p = 0.0021) and mock Exam 2 (66% vs. 58%; β = 8.95, 95% CI [3.54, 14.37], p = 0.0015). Regarding ABSITE performance, IMGs obtained significantly higher scores compared to nIMGs (560 vs. 505; β = 70.3, 95% CI [17.26, 123.34], p = 0.010). This trend was consistent when analyzing ABSITE percentages (74% vs. 68%; β = 7.93, 95% CI [2.16, 13.7], p = 0.0077).

**Table 1:** Comparison of Quiz %, Exam Scores, and ABSITE Performance Between International and Non-international Medical Graduates Using Linear Mixed Models nIMG: non-international medical graduates; IMG: international medical graduates; ABSITE: American Board of Surgery In-Training Examination. Data presented as percentages ± standard deviations.

Outcomes	Overall (N = 48)	nIMG (N = 24)	IMG (N = 24)	Beta (95% CI)	p-value
Quiz % ±SD	65±10	61±10	67±9	7.29 (2.58, 12)	0.0029
Exam 1 ±SD	62±10	58±10	64±9	7.43 (2.79, 12.06)	0.0021
Exam 2 ±SD	63±13	58±11	66±13	8.95 (3.54, 14.37)	0.0015
ABSITE Score ±SD	536±98	505±104	560±86	70.3 (17.26, 123.34)	0.010
ABSITE % ±SD	72±10	68±11	74±9	7.93 (2.16, 13.7)	0.0077

Analysis of quiz percentages, mock exam scores, and ABSITE performance was conducted between DO and MD participants (Table 2). Among the 48 participants, six held DO degrees, while 42 held MD degrees. There were no statistically significant differences between DO and MD participants in quiz percentages (62% vs. 65%; β = 4.31, 95% CI [-3.45, 12.07], p = 0.27), mock Exam 1 scores (57% vs. 62%; β = 6.72, 95% CI [-0.72, 14.17], p = 0.076), mock Exam 2 scores (60% vs. 64%; β = 2.74, 95% CI [-6.57, 12.05], p = 0.56), ABSITE scores (498 vs. 542; β = 46.12, 95% CI [-39.33, 131.57], p = 0.29), and ABSITE percentages (68% vs. 72%; β = 5.37, 95% CI [-3.94, 14.69], p = 0.25).

**Table 2:** Comparison of Quiz %, Exam Scores, and ABSITE Performance Between Doctor of Osteopathic Medicine and Doctor of Medicine Using Linear Mixed Models DO: doctor of osteopathic medicine; MD: doctor of osteopathic medicine; ABSITE: American Board of Surgery In-Training Examination. Data presented as percentages ± standard deviations.

Outcomes	Overall (N = 48)	DO (N = 6)	MD (N = 42)	Beta (95% CI)	p-value
Quiz % ±SD	65±10	62±8	65±10	4.31 (-3.45, 12.07)	0.27
Exam 1	62±10	57±9	62±10	6.72 (-0.72, 14.17)	0.076
Exam 2	63±13	60±10	64±13	2.74 (-6.57, 12.05)	0.56
ABSITE Score	536±98	498±59	542±101	46.12 (-39.33, 131.57)	0.29
ABSITE %	72±10	68±6	72±11	5.37 (-3.94, 14.69)	0.25

Linear mixed model analysis was used to investigate the influence of IMGs and MD degrees on ABSITE Performance (Table 3). The analysis revealed notable differences in ABSITE scores and percentages between IMGs and nIMGs. IMG residents demonstrated significantly higher ABSITE scores (β = 70.3, 95% CI [17.26, 123.34], p = 0.010) and percentages (β = 7.93, 95% CI [2.16, 13.7], p = 0.0073) compared to their nIMG counterparts.

**Table 3:** Investigating the Influence of International Medical Graduates and Doctor of Medicine Degrees on ABSITE Performance Using Linear Mixed Models ABSITE: American Board of Surgery In-Training Examination

Predictors	ABSITE Score		ABSITE %	
	Beta (95% CI)	p-value	Beta (95% CI)	p-value
International Medical Graduates vs. Non-international Medical Graduates	70.3 (17.26, 123.34)	0.010	7.93 (2.16, 13.7)	0.0073
Doctor of Medicine vs. Doctor of Osteopathic Medicine	46.12 (-39.33, 131.57)	0.29	5.37 (-3.94, 14.69)	0.25

Regarding the type of medical degree, there were no statistically significant differences observed between residents holding MD and DO degrees. Both groups showed similar ABSITE scores (β = 46.12, 95% CI [-39.33, 131.57], p = 0.29) and percentages (β = 5.37, 95% CI [-3.94, 14.69], p = 0.25).

## Discussion

The findings of this study contribute valuable insights into the performance differentials observed among general surgery residents, particularly regarding their performance on the ABSITE. ABSITE scores play a pivotal role in shaping the trajectories of surgical careers, influencing opportunities for fellowship placements and career advancement [5]. Our analysis revealed notable disparities in ABSITE performance between IMGs and nIMGs, with IMGs demonstrating significantly higher scores and percentages compared to their nIMG counterparts. This finding aligns with previous research indicating variations in educational backgrounds and training experiences among IMGs, which may contribute to differences in examination performance [6].

Furthermore, the comparison of ABSITE performance between MD and DO participants yielded intriguing insights. Despite concerns regarding potential disparities in educational curriculum and clinical training between MD and DO programs, our analysis found no statistically significant differences in ABSITE scores or percentages between the two groups. This suggests that, within the context of our study population, both MD and DO graduates exhibited comparable levels of preparedness and proficiency in surgical knowledge and skills, as assessed by the ABSITE. 

Our study's cohort included only six (12%) DO graduates. This aligns with broader trends showing significantly lower match rates for DO candidates compared to MD candidates, particularly in competitive surgical subspecialties [7]. This disparity can be attributed partly to fewer program directors ranking DO applicants and a lack of emphasis on osteopathic principles in the residency application process [7]. Additionally, differences in clerkships and experience play a role [8].

In a related study, MDs were found to have longer clerkships, with more home sub-internships compared to DOs [8]. While there was no discernible difference in self-reported confidence levels in knowledge or technical skills between the two groups, DOs expressed less confidence in their medical school preparation and were more likely to perceive inequality in residency preparation [8]. Addressing these disparities may involve increasing advocacy efforts at both local and national levels, enhancing mentorship programs, providing greater exposure to surgical subspecialties for DO medical students, and ensuring greater involvement of selected surgical subspecialties in teaching and supporting diverse DO applicants. These efforts are crucial not only for strengthening the medical field but also for addressing the predicted physician shortages. Moreover, the absence of significant differences in ABSITE performance between MD and DO graduates highlights the merit of both educational pathways in preparing surgeons for the rigors of clinical practice and board certification examinations.

Our research highlights the intricate nature of surgical education and training, which is shaped by various factors including the structure of medical school curricula, residency programs, and individual learning experiences [6,9]. To practice as an IMG physician or resident in the US, one must meet stringent requirements established by the Educational Commission for Foreign Medical Graduates (ECFMG), ensuring the equivalence of qualifications for IMGs [6,9]. It's noteworthy that some IMG residents in the US continue to practice as physicians overseas [6,9]. The notable performance of IMGs on the ABSITE may stem from their diverse perspectives, backgrounds, and clinical exposures gained through international medical training. This enriches the educational environment of surgical residency programs and promotes cross-cultural exchange [6,9].

Moreover, future research could explore whether residents who performed poorly on the ABSITE match into their preferred specialties and successfully pass their board examinations. Previous studies have indicated that residents who demonstrate inadequate performance on the ABSITE at any point during their residency are at a heightened risk of failing both their ABS qualifying and certifying examinations [10]. Investigating the long-term outcomes of residents with suboptimal ABSITE scores could provide valuable insights into the predictive validity of this examination and inform strategies for supporting residents' academic success and career advancement. Additionally, implementing weekly educational programs with assigned reading and examinations may offer a proactive approach to improving ABSITE performance and increasing first-time pass rates for the ABS qualifying exam [11].

Limitations/future studies

Acknowledging the constraints of our study is crucial, given its retrospective design, reliance on data from a single institution, and potential unaccounted confounding variables. Future research should delve into the mechanisms behind performance variations on exams like the ABSITE, considering educational methods, clinical exposure, and cultural influences. Longitudinal studies tracking residents' progress could offer insights into predictors of surgical competency.

While our study contributes significantly, it has limitations. The sample size was small and limited to general surgery residents from one institution and one timeframe, reducing generalizability. Additionally, focusing solely on ABSITE scores overlooks other factors affecting surgical competency, like clinical skills and procedural experience. The retrospective nature might introduce bias or unaccounted variables. Furthermore, we didn't evaluate the impact of interventions to enhance ABSITE performance, warranting further investigation into effective training strategies. Furthermore, certain factors influencing the outcomes of test days have been overlooked. For instance, a study noted that residents who took a vacation in January before the examination, despite showing no apparent difference in baseline test-taking ability, scored higher on the ABSITE [12]. This study is constrained by its focus on only one general surgery program. However, previous research has underscored the significance of program-related factors. Specifically, programs where Program Directors actively participated in remediation mentorship and monitored residents' reading habits achieved higher ABSITE percentile scores. Additionally, programs that implemented a lower ABSITE threshold for remediation demonstrated improved performance on the examination [13]. Additionally, other data variables, including age distribution, gender, educational background, healthcare experience, and the United States Medical Licensing Examination score (USMLE score) were not considered in this review. Future studies should investigate these variables to determine if any relationships exist. Additional limitations of this study involve incorporating both preliminary and categorical general surgery residents throughout all five years of residency. Future investigations may improve by excluding preliminary residents and examining each year of residency separately. At Marshall University, TrueLearn is seamlessly integrated into the educational curriculum, eliminating extra fees for residents and simplifying accessibility. This model could be adopted by other programs to promote resident engagement.

Further research could build on our findings by exploring various aspects of surgical education and training. Longitudinal studies could shed light on ABSITE performance's long-term effects on career paths and patient outcomes. Qualitative methods could uncover factors influencing exam performance, like educational experiences and support systems. Comparative studies across healthcare systems and cultures could provide insights into training methodologies' effectiveness. Prospective studies could assess interventions like simulation-based training to optimize surgical education.

## Conclusions

In conclusion, our study sheds light on the performance differentials observed among general surgery residents on the ABSITE. The findings underscore the importance of understanding the factors influencing examination performance, including educational backgrounds, training experiences, and cultural influences. By elucidating these disparities, our study contributes to ongoing efforts to enhance surgical education, training methodologies, and workforce diversity within the field of general surgery. Through targeted interventions and collaborative initiatives, we can strive to foster inclusivity, equity, and excellence within surgical residency programs, ultimately ensuring the development of a diverse, competent, and resilient cadre of surgical practitioners capable of meeting the evolving challenges of modern healthcare delivery.
